# Nature Is a Valuable Source for the Biosynthesis of Nanoparticles and Their Effects on the Treatment of Osteomyelitis

**DOI:** 10.1111/jcmm.71003

**Published:** 2025-12-25

**Authors:** Amir Reza Sadeghifar, Alireza Farsinejad, Naghmeh Satarzadeh, Arman Shahabi, Amin Sadeghi Dousari

**Affiliations:** ^1^ Department of Orthopedics, School of Medicine Kerman University of Medical Sciences Kerman Iran; ^2^ Stem Cells and Regenerative Medicine Innovation Center Kerman University of Medical Sciences Kerman Iran; ^3^ Department of Hematology and Medical Laboratory Sciences, Faculty of Allied Medicine Kerman University of Medical Sciences Kerman Iran; ^4^ Student Research Committee Kerman University of Medical Sciences Kerman Iran

**Keywords:** biosynthesis, green synthesis, nanoparticles, osteomyelitis

## Abstract

Infection is still one of the biggest threats to global health. Today, treating infections such as osteomyelitis is challenging due to the rise in drug‐resistant and biofilm‐forming pathogens. Therefore, researchers worldwide are seeking new ways to combat these infections. Nanotechnology, which is of interest in the medical field, has provided a platform for drug delivery and the treatment of osteomyelitis. Various biological, chemical, and physical methods are used for the biosynthesis of nanoparticles. Among these methods, biological methods, or green synthesis, are of great interest due to their non‐toxicity, high stability, low cost, and environmental friendliness. This study aimed to investigate nanoparticles biosynthesised from natural sources and use them for the treatment of osteomyelitis.

## 1 Introduction

Osteomyelitis, an inflammation of the bone and bone marrow caused primarily by a microbial pathogen, is an increasing health problem [1, 2, 3]. In approximately 75% of cases, osteomyelitis is caused by various species of *Staphylococcus*, with *Staphylococcus aureus* being the most common cause (30% to 60%) [4, 5]. This disease can involve one or more tissues and locations within the bone, including the periosteum, overlying soft tissues, cortex, bone marrow, and spongy bone [6]. The major types of osteomyelitis in adults include diabetic foot infection (DFI; associated with peripheral vascular disease common in diabetes), periprosthetic joint infection (PJI; associated with orthopaedic implants), and fracture‐related infection (FRI; associated with open fractures). Due to factors such as population aging, rising rates of vascular and metabolic diseases, and increased demand for joint replacement surgery, the prevalence of osteomyelitis is increasing [7].

Although several treatment protocols are based on staging and diagnostic criteria, osteomyelitis remains challenging to treat [2]. This is because many individuals are affected by recurrent infections, one of which involves biofilm production by staphylococcal pathogens. Staphylococcal biofilms reduce oxygen and vascular permeability by forming and growing within bone [8]. Therefore, antibiotic treatments do not effectively penetrate the internal source of infection or the biofilm matrix, allowing the infection to spread and necessitating extensive debridement procedures and possible amputation. In addition, increased cytokine production due to infection increases osteoblast cell death and compromises bone formation [9]. Taken together, these factors result in a bone that is prone to fracture and subsequent injury [8].

Traditional treatments for osteomyelitis include surgery to remove dead tissue and systemic antibiotics, but these methods have significant limitations. The failure rate of conventional clinical treatments is due to inadequate drug delivery to the site of infection, significant microbial resistance, especially in resistant bacteria such as MRSA, long treatment courses with severe side effects, and a high risk of relapse [10]. Despite these problems, researchers are seeking new treatments with therapeutic potential, including nanotechnology, which has attracted attention from many researchers worldwide [11].

Nanoscience has enabled significant advances across various fields, including engineering and medicine [12, 13, 14]. Among nanomaterials, metal nanoparticles have attracted the most attention among researchers due to their high potential and diverse applications. Among the properties that make metal nanoparticles valuable are the increased number of reactive sites, the high surface area‐to‐volume ratio, and thermal and mechanical stability [15, 16, 17]. Various types of metal nanoparticles and metal oxides have shown a wide range of applications, including pharmaceuticals, biomedicine, and biosensors [18, 19].

Various methods for synthesising nanoparticles include biological, chemical, and physical methods [20, 21, 22, 23, 24]. Physical and chemical methods have disadvantages, such as high energy consumption and the use of toxic and hazardous materials, which have led to the growing prominence of biological methods, or green synthesis, as an alternative among researchers worldwide [25, 26, 27, 28, 29].

Green synthesis methods offer many advantages, such as biocompatibility, ease of synthesis, low cost, and high production efficiency [30, 31]. In green synthesis methods, fungi, yeasts, bacteria, microalgae, and plants are used for biosynthesis [32, 33, 34]. Plant extracts, which include fruits, leaves, roots, stems, and flowers, are a significant source for the biosynthesis of nanoparticles. Also, other materials found in nature, such as plant gums, remains of various organisms, and other environmentally friendly materials, are used for the biosynthesis of nanoparticles [35, 36, 37].

Given that several studies have investigated the use of various biosynthesised nanoparticles in nature for the treatment of osteomyelitis, this review aimed to examine their effects.

## 2 Characterisation of Biosynthesised Nanoparticles With Naturally Occurring Substances Effective on Osteomyelitis

**FIGURE 1:**
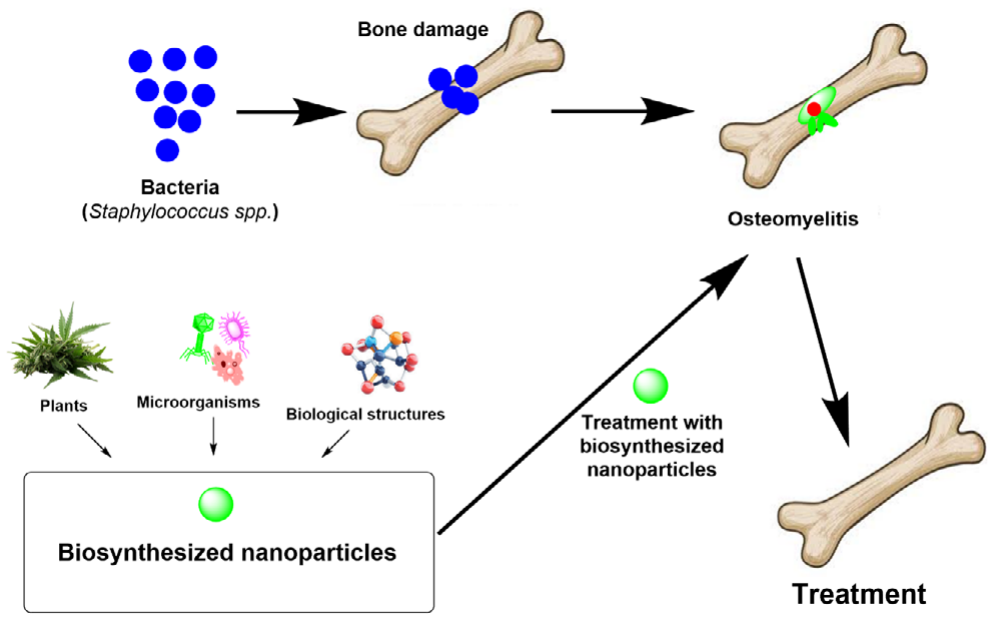
Schematic image of the effects of biosynthesised nanoparticles on osteomyelitis.

Several studies have biosynthesised various nanoparticles using naturally available sources and investigated their effects on osteomyelitis (Table 1). These studies used different sources, such as plants ( *Azadirachta indica* , *Coccinia grandis* ), sesame seed oil, cockle shells (*Anadara granosa*), gellan gum (produced by *Sphingomonas elodea*), silk fibroin, bone cement, and hydroxyapatite, for the biosynthesis of nanoparticles. The most biosynthesised nanoparticles were silver, and other nanoparticles, including hydroxyapatite, magnetite, SF, PLGA, and CaCO_3_, were also biosynthesised. Various analyses, such as FTIR, UV–Vis, DLS, XRD, TEM, ED, FE‐SEM, zeta potential, AFM, and SEM, were used to investigate the properties of nanoparticles. SEM and DLS analysis showed that nanoparticle sizes ranged from 35 to 258 nm across different studies, and TEM analysis showed that most nanoparticles were spherical. However, other shapes, such as hexagonal and cubic, have also been reported. Biosynthesised nanoparticles derived from naturally occurring sources showed strong potential for treating osteomyelitis, as discussed below (Figure 1).

**TABLE 1:** Characterisation of metal nanoparticles biosynthesised affecting osteomyelitis.

Origin of biosynthesis	Nanoparticle	Structure	Analyzes performed	Size (nm)	Shape	Applications	Refs.
Silk fibroin	Ag	—	FTIR	—	—	AgNPs helped to resolve abscesses and inflammation, and also helped to produce fibrosis and neovascularization	[38]
Hydroxyapatite	HA‐Fe	HA‐Fe_3_O_4_	SEM, EDS	—	—	HA‐Fe NPs were biocompatible with an absorption time of more than 90 days. HA‐Fe NPs can be used as a bone implant	[39]
Silk fibroin	SF	VANCO‐ SF	DLS, FESEM, FTIR	80–90	Spherical	NPs caused osteomyelitis infections to be treated at the defect site with better results than other control groups	[40]
Gellan gum (produced by *Sphingomonas elodea*)	PLGA	VANCO‐PLGA	DLS, AFM	258 ± 11	Spherical	NPs with antibacterial activity inhibited the growth of standard *Staphylococcus* spp. as well as clinical strains isolated from osteomyelitis joints NPs are compatible with osteoblast‐like MG‐63 cells	[41]
Cockle shells (*Anadara granosa*)	CaCO_3_ (Aragonite)	VANCO‐CaCO_3_	TEM, zeta potential	~35	Cubic	NPs had a high antibacterial effect against methicillin‐resistant *S. aureus ATCC 29213* and also the cell proliferation assay showed 80% cell viability of the embryonic osteoblast cell line treated	[42]
Bone cement	Ag	Ag‐TTCP‐DCPD	—	< 100	—	NPs did not cause any cytotoxicity NPs showed antibacterial effects by creating a zone of inhibition between 1.4 and 13.3 mm NPs in the study of mice, the number of bacterial colonies in the 12‐week nanoparticle treatment groups was reduced compared to the 3‐week groups	[43]
Sesame seed oil	Ag	—	UV–Vis	—	—	NPs produced a 23 mm inhibition zone against *S. aureus*	[44]
*Azadirachta indica* , *Coccinia grandis*	HA	—	FTIR, XRD, TEM	~53	Hexagonal	Biosynthesised HANPs produced a 26 mm inhibition zone against *S. aureus* and *E. coli*	[45]

## 3 Effects of Biosynthesised Nanoparticles With Naturally Occurring Materials on Osteomyelitis

In a study, Rong et al. [38] investigated the effects of silver nanoparticles biosynthesised by silk fibroin on traumatic osteomyelitis of extremities (TOE). The treatment of TOE has become difficult due to the emergence of multidrug‐resistant strains, such as methicillin‐resistant *Staphylococcus aureus* (MRSA). In this study, the Kirby‐Bauer inhibition zone method was used to investigate the antibacterial effects of silver nanoparticles at different concentrations, alone and in combination with gentamicin, against this bacterium. Osteoblast cells, including MC‐3T3 cells and mouse bone cells, were also used to assess cell growth in response to different concentrations of silver nanoparticles. The results of this study showed that the combination of silver nanoparticles in patients helped resolve abscesses and inflammation, and also promoted fibrosis and neovascularization. Overall, biosynthesised silver nanoparticles from silk fibroin can serve as a new platform for the treatment of TOE.

In another study, Ferreira‐Ermita et al. [39] investigated the effects of biosynthesised magnetite nanoparticles with hydroxyapatite (HA‐Fe_3_O_4_) as a bioactive agent on the treatment of osteomyelitis. In this study, six New Zealand white rabbits were used to investigate the biocompatibility, and four samples of the nanoparticle–hydroxyapatite combination were implanted between muscle tissue and fascia in each rabbit. Then, biopsy samples, including tissue and implant samples, were collected at 15, 30, 60, and 90 days for analysis. The results of this study showed that the desired combination was biocompatible and maintained an absorption time exceeding 90 days. It was also shown that the desired combination can be used as a bone implant due to its specific microtopographic surface and morphological and structural characteristics. In general, the magnetite nanoparticle–hydroxyapatite combination is a suitable option for bone regeneration in cases of osteomyelitis infections.

In another study, the effects of biosynthesised nanoparticles with silk fibroin as a carrier for vancomycin in the treatment of severe bone infection in a rat tibia osteomyelitis model were investigated. In this study, SFNPs were used as carriers of the antibiotic vancomycin to provide sustained drug delivery. Also, the release kinetics of vancomycin alone and in combination with SFNP scaffolds were investigated at two different pH values (4.5 and 7.4). The antibacterial activity of the desired compound against the pathogen methicillin‐resistant *S. aureus* (MRSA), which causes osteomyelitis (injection of 8 × 10^6^ CFU of MRSA into the tibia of the mouse), was investigated at two pH values using the disk diffusion method. After histopathological and radiographic evaluation of the compound's effectiveness at 6 weeks, results showed that osteomyelitis at the defective site was reduced, with better outcomes than in other treatment groups [40].

Posadowska et al. [41] investigated the effects of biosynthesised nanoparticles with gellan gum (a polysaccharide, produced by anaerobic fermentation of carbohydrates by *Sphingomonas elodea*) as a carrier for the antibiotic vancomycin for the treatment of osteomyelitis. In this study, vancomycin was encapsulated within poly(L‐lactide‐co‐glycolide) nanoparticles, yielding an injectable composition. The MG‐63 cell line was also used to investigate compatibility. The results of this study showed that the nanoparticles with antibacterial activity inhibited the growth of standard *Staphylococcus* spp. and clinical strains isolated from osteomyelitis joints. It was also compatible with osteoblast‐like MG‐63 cells. Overall, the biosynthesised PLGA nanoparticles demonstrated tunable drug release, self‐repair after disruption, and antibacterial activity against osteomyelitis infections.

Saidykhan et al. [42] investigated the effects of aragonite nanoparticles biosynthesised with cockle as a vancomycin drug carrier for the treatment of osteomyelitis. The results of this study showed that the nanoparticles exhibited a strong antibacterial effect against methicillin‐resistant *S. aureus* ATCC 29213 after 5 days. Also, the cell proliferation assay showed 80% cell viability in the embryonic osteoblast cell line treated with the highest VANP concentration (250 μg/mL), indicating good biocompatibility of these nanoparticles.

In a study, Suk Choi et al. [43] investigated the effects of biosynthesised silver nanoparticles combined with bone cement composed of tetracalcium phosphate–dicalcium phosphate dihydrate (TTCP) on osteomyelitis. This study investigated the cytotoxic effects of the Ag–TTCP–DCPD nanoparticles on osteocyte and fibroblast cell lines. They also investigated Ag–TTCP–DCPD NPs at varying concentrations for 3 to 12 weeks in a mouse model of tibial osteomyelitis caused by methicillin‐resistant *S. aureus* . In addition, they evaluated the antibacterial activity of these nanoparticles using the disk diffusion method against methicillin‐resistant *S. aureus* . The results of this study showed that different concentrations of Ag–TTCP–DCPD NPs did not cause any cytotoxicity. The nanoparticles exhibited antibacterial activity, producing a zone of inhibition ranging from 1.4 to 13.3 mm. Also, in the mouse study, the number of bacterial colonies in the 12‐week nanoparticle treatment groups was lower than in the 3‐week groups. Overall, the results showed that silver nanoparticle bone cement (Ag–TTCP–DCPD NPs) can be considered for the treatment of osteomyelitis.

Sudhakar et al. [44] investigated the effects of biosynthesised nanoparticles derived from sesame seed oil on the treatment of osteomyelitis. In this study, biosynthesised nanoparticles and also the antibiotic amoxicillin were tested on *S. aureus* , which causes osteomyelitis, using the disk diffusion method. The results of this study showed that biosynthesised silver nanoparticles produced a 23 mm inhibition zone against *S. aureus* , while the antibiotic amoxicillin produced a 25 mm inhibition zone. This study shows that biosynthesised silver nanoparticles have high potential for treating osteomyelitis.

In another study, Kumar et al. [45] investigated the effects of biosynthesised hydroxyapatite (HA) nanoparticles on the treatment of osteomyelitis. In this study, biosynthesised nanoparticles were tested on *E. coli* and *S. aureus* using the disk diffusion method. The results of this study showed that biosynthesised HANPs produced an inhibition zone of 26 mm against *S. aureus* and *E. coli* . Overall, green synthesis could be a promising approach for developing orthopaedic biomaterials with antibacterial properties.

## 4 Future Development Trends

Given recent advances in nanotechnology, future approaches to treating osteomyelitis will focus on developing biocompatible, smart nanoparticles that not only possess high antibacterial activity but also can precisely target damaged tissues and control drug delivery. The use of natural nanocomposites and nanopolymers with biodegradable properties and the ability to activate biological responses is expected to be an effective trend for improving current treatments. Also, the development of multi‐targeted drug delivery systems and magnetic nanoparticles to increase treatment efficiency and reduce side effects will be among the most important research priorities in the future. These trends can not only improve patients' quality of life but also reduce microbial resistance and prolong therapeutic effects.

## 5 Conclusions

Nowadays, the use of nanoparticles in medicine and disease treatment has attracted the attention of researchers worldwide. Therefore, this study examined various nanoparticles biosynthesised from natural sources and their effects on osteomyelitis. The results of this study showed that nanoparticles biosynthesised from biological sources, such as silver magnetite and hydroxyapatite, have high potential for treating osteomyelitis due to their biocompatibility and antibacterial properties. This study also showed that these biosynthesised nanoparticles can be used as drug carriers for the treatment of osteomyelitis.

## Author Contributions

**Amir Reza Sadeghifar:** conceptualization (equal). **Alireza Farsinejad:** conceptualization (equal). **Naghmeh Satarzadeh:** conceptualization (equal). **Arman Shahabi:** conceptualization (equal). **Amin Sadeghi Dousari:** conceptualization (equal).

## Conflicts of Interest

The authors declare no conflicts of interest.

## Data Availability

Data sharing not applicable—no new data generated, or the article describes entirely theoretical research.
